# Factors Associated with Pregnancy Intentions Amongst Postpartum Women Living with HIV in Rural Southwestern Uganda

**DOI:** 10.1007/s10461-018-2317-9

**Published:** 2018-10-26

**Authors:** Esther C. Atukunda, Godfrey R. Mugyenyi, Elly B. Atuhumuza, Angella Kaida, Adeline Boatin, Amon G. Agaba, Lynn T. Matthews

**Affiliations:** 1 Mbarara University of Science and Technology, Mbarara, Uganda; 2 Faculty of Health Sciences, Simon Fraser University, Burnaby, Canada; 3 Massachusetts General Hospital and Harvard Medical School, Boston, MA USA; 4 Division of Infectious Diseases and Center for Global Health, Massachusetts General Hospital, Boston, MA USA

**Keywords:** Family planning, Pregnancy desire, WLWH, Postpartum contraception, Uganda

## Abstract

Comprehensive HIV treatment and care makes it safer for women living with HIV (WLWH) to have the children they desire, partly through provision and appropriate use of effective contraception. However, nearly one third of WLWH in-care in a large Ugandan cohort became pregnant within 3 years of initiating ART and half of these incident pregnancies (45%) were unplanned. We therefore describe future pregnancy plans and associated factors among postpartum WLWH in rural southwestern Uganda in order to inform interventions promoting postpartum contraceptive uptake. This analysis includes baseline data collected from adult WLWH enrolled into a randomized controlled trial to evaluate the effect of family planning support versus standard of care at 12 months postpartum in southwestern Uganda. Enrolled postpartum WLWH completed an interviewer-administered questionnaire at enrolment. Among 320 enrolled women, mean age, CD4 count, and duration on ART was 28.9 (standard deviation [SD] 5.8) years, 395 cells/mm^3^ (SD = 62) and 4.6 years (SD = 3.9), respectively. One-hundred and eighty nine (59%) of women reported either personal (175, 55%) or partner (186, 58%) desire for more children in the next 2 years. Intentions to have more children was strongly associated with partner’s desire for more children (AOR = 31.36; P < 0.000), referent pregnancy planned (AOR = 2.69; P = 0.050) and higher household income > 150,000 Shs per month (AOR = 1.37; P = 0.010). Previous use of modern contraception (AOR = 0.07; P = 0.001), increasing age (AOR = 0.34; P = 0.012), having > 2 own children living in a household (AOR = 0.42; P = 0.021) and parity > 2 (AOR = 0.59; P = 0.015) were associated with reduced odds of pregnancy intention. Our findings highlight the role male partners play in influencing pregnancy intentions postpartum and the importance of engaging men in sexual and reproductive health counselling about child spacing for the health of women, children, and families. This should be addressed alongside key individual-level social, demographic, economic and structural factors within which couples can understand risks of unplanned pregnancies and access effective contraceptive methods when they need or want them.

## Introduction

The second prong of the WHO strategy to eliminate mother to child transmission of HIV (EMTCT) is to prevent unplanned pregnancies for women living with HIV (WLWH). HIV treatment and care makes it safer for WLWH to have the children they desire, while provision and appropriate use of effective contraception are important strategies to prevent unintended pregnancies. There remains an unmet need for family planning among WLWH in Uganda [1], where the modern contraceptive prevalence rate for married women overall is 35% [2]. Less than half of sexually-active WLWH accessing ART in a large Ugandan cohort used effective contraception, of which 44% relied on male condoms [3].

Pregnancy incidence of 9.40 per 100 women years has been reported within 4 years following ART initiation amongst WLWH in Uganda [4]. Almost half of these incident pregnancies (45%) were unplanned [5]. In this same cohort, unplanned pregnancy was not associated with subsequent effective contraceptive use postpartum, with only half of the women with unplanned pregnancies and 44% with planned pregnancies reporting effective contraception postpartum. Other studies report up to 85% of pregnancies occurring within 3 years following ART initiation as unwanted [6]. Although increasing desires to have children are reported amongst mutually-disclosed serodiscordant couples in Uganda [7], couples often disagree in their perceptions of each partner’s plans for pregnancy, understandings of HIV transmission, and acceptable level of HIV risk to meet their reproductive goals [8]. While supported pregnancies are an important part of life for many HIV-affected couples, unwanted and/or unplanned pregnancies can lead to poor maternal and child outcomes including perinatal HIV transmission, pregnancy complications, and increased economic burden of care for self and others among others.

Previous studies have investigated individual and partner-level predictors collected prior to pregnancy, including pregnancy desire [1], and contraception uptake [3, 5]. However, understanding individual and or partner characteristics that influence pregnancy intentions in the immediate postpartum period among WLWH may be important to informing policy, promoting contraceptive counselling and uptake postpartum. In this paper, we examine individual and partner characteristics associated with pregnancy intentions in the next 2 years amongst recently postpartum WLWH delivering at a large clinical center in rural southwestern Uganda. We also describe the history, patterns and perception of prior contraception use. These data may inform integrated programs to support WLWH by providing counselling and contraception access for postpartum women.

## Materials and Methods

### Study Design and Setting

This analysis includes baseline data collected from WLWH enrolled in a randomized controlled trial in southwestern Uganda. The parent trial aims to evaluate the effect of family planning support versus standard of care on contraceptive use at 12 months postpartum (NCT02964169). All study procedures were conducted at the Mbarara Regional Referral Hospital (MRRH), a publicly-funded teaching hospital in rural southwestern Uganda serving 10 districts with a population of over 5 million people. The hospital delivers over 12,000 babies annually, with a maternal HIV prevalence of 10.2% (MRRH records).

### Participants and Recruitment

This study was initiated in October 2016 and enrolment ended in May 2017. Follow-up of participants is ongoing. Eligible participants were WLWH women ≥ 18 years of age, admitted in a postnatal ward at MRRH within 5 days postpartum regardless of pregnancy outcome and qualified for any family planning methods available. The exclusion criteria included: (1) HIV negative, (2) history of hypersensitivity to latex, (3) no male sexual partner and/or not anticipating one for the next 2 years, (4) only sexual partner has had vasectomy and (5) inability to complete informed consent process as assessed by the study nurses. Trained research assistants (RAs) approached WLWH in postnatal ward at least 12 h after delivery.

RAs obtained voluntary written informed consent from all eligible participants. All consenting participants gave written informed consent, or for those who could not write, a thumbprint was made on the consent form.

### Study Groups and Data Collection

We screened 378 WLWH and enrolled a total of 320 who were equally randomized into the intervention arm (Family planning support) and standard of care (control group) between October 2016 and May 2017. These women are being followed for 1 year. All participants completed baseline interviewer-administered interviews and phlebotomy for CD4 cell count and to confirm HIV sero-status. Interviews were conducted by two trained research assistants fluent in English and the main local language in a private office space. Each interview took about 30–45 min. Data was collected electronically. A transport refund of $3 was given on each visit.

### Study Measures

The primary outcome of interest, pregnancy intention in the next 2 years, was assessed using the CDC Pregnancy Risk Assessment Monitoring System Instrument [9–11]. This particular question was asked in two ways, (1) through a Linkert scale (5-point) asking women to agree or disagree with a given statement, “I still want to give birth to more children in the next 2 years”. To create a binary response, agree or strongly agree was coded as “yes” while all other responses, including “neither agree or disagree”, were coded as “no”. A second question was, “Would you like to have another child/children in the next 2 years?” with an expected response of yes/no. Regression analysis of both responses from the two questions generated identical outcomes, thus confirming the internal validity and consistency of the two measures. For the current analysis, we used responses for the direct question, “would you like to have another child/children in the next 2 years?” as our primary outcome of interest referred to as “pregnancy intention”.

A blood sample was drawn at baseline to confirm the HIV status and measure CD4 cell count. A structured face-to-face questionnaire was completed at enrollment to collect information on socio-demographics, depression, health [12], reproductive history, partnership dynamics (e.g. HIV serostatus disclosure, partner HIV-serostatus), perception, use and knowledge of contraception, decision making [4, 6, 13–18], food insecurity [19, 20], alcohol use in the last 9 months [21], HIV stigma [22], and social support [23]. A primary partner was defined either as the “main partner”, who is also a regular sexual partner, or the most recent sexual partner if no main partner was named. Modern family planning was defined as use of contraceptive pills, male/female Condoms, diaphragm, cervical cap, intrauterine device (IUD), contraceptive implant, injectables & emergency contraception methods to limit or space the number of children one would wish to have.

### Statistical Analysis

We describe demographic and clinical data for the cohort using standard descriptive statistics. We assessed the prevalence and covariates of reporting pregnancy intentions in the next 2 years. The Household Food Insecurity Access Scale (HFIAS) was calculated as recommended [24]. Univariable logistic regression was used to assess unadjusted associations between covariates and pregnancy intentions, expressed using crude odds ratio and 95% confidence intervals. Variables with p value ≤ 0.10 in unadjusted analyses were considered for inclusion in a multivariable logistic regression analysis. Variables examined in the unadjusted model found to be collinear were selectively excluded from the multivariate models or added one at a time to observe their respective effect. A sub-analysis to establish the effect of partner pregnancy intentions was also done. Statistical significance was defined at the level of p ≤ 0.05. All data analyses were performed using STATA version 12.0 (Statacorp, College Station, Texas, USA).

#### Ethical Approval

This study was approved by the Institutional Review Council of Mbarara University of Science and Technology and Uganda National Council of Science and Technology, and registered with clinicaltrials.gov (NCT02964169).

## Results

### Participant Characteristics

A total of 320 participants were randomized and enrolled equally into the family planning support and control arms of the study following delivery at MRRH. The mean age of participants was 28.9 (Standard Deviation [SD] 5.8 years). Almost half of participants (46%) attained education greater than primary. All women were accessing ART with a mean duration of 4.5 years (SD = 3.9) and a mean CD4 count of 395 cells/mm^3^ (SD = 62). Majority of women (N = 266, 83%) reported planning their most recent pregnancy. Most (N = 214, 67%) participants had attended at least four prenatal visits in their referent pregnancy. Most women (N = 281, 88%) had disclosed their HIV-serostatus to their sexual partners or spouses. Ninety women (28%) reported household income of more than 150,000 Ugandan Shilling (~ 40 USD) per month. The majority reported living within monogamous households (N = 265, 83%). Almost half of the participants (43%) reported at least three children living within the household with 58% reporting three or more prior pregnancies. About 19% (N = 61) of participants experienced severe food insecurity while HIV stigma was moderately common amongst the study participants (Median score = 4 [IQR = 2–6]). Alcohol consumption in the past 9 months was reported by 68% (N = 216). Fewer participants (33%) received moderate to adequate social support (median score = 2.4 [IQR = 2.1–2.8]). Other baseline characteristics are presented in Table 1.

**Table 1:** Baseline demographic and clinical characteristics of recently postpartum women living with HIV in Uganda, N = 320

Characteristics	Mean (SD) or n (%)
Mean age (years)	28.9 (5.8)
Partner age (years)	34.7 (7.2)
Educational attainment greater than primary	148 (46.3)
Mean duration on ART (years)	4.5 (3.9)
Mean CD4 (SD)	395 (62)
Children < 18 years in household, median (IQR)	2 (1.3)
Parity	
1	54 (16.9)
2	80 (25.0)
3	84 (26.3)
4	56 (17.5)
≥ 5	46 (14.3)
Prenatal visits attended	
1	10 (3.1)
2	19 (5.9)
3	77 (24.1)
4	148 (46.3)
≥ 5	66 (20.6)
Severe food insecurity^a^	61 (19.1)
HIV stigma^b^, median (IQR)	4 (2–6)
Depression score^c^, median (IQR)	5 (3–10)
Consume alcohol in the last 9 months	216 (67.5)
Median social support score^d^, (IQR)	2.4 (2.1–2.8)
Member of support group	149 (46.6)
Household income > 150,000^e^ Shs per month	90 (28.1)
Household description	
Single householder	19 (5.9)
Monogamous household	265 (82.8)
Polygamous household	36 (11.2)
At least one adult working in a household	247 (77.2)
Vaginal mode of delivery for last pregnancy	254 (79.4)
Most recent pregnancy was planned	266 (83.1)
Disclosed HIV sero-status to sexual partner/spouse	281 (87.8)

^a^HFIAS > 8 means severe food insecurity

^b^This score ranges from 1 to 8, with 8 indicating high levels of HIV stigma

^c^This score ranges from 1 to 48 indicating 0 as no depression

^d^This score ranges from 1 to 4, with 4 indicating high levels of social support

^e^An equivalent to about 40 USD

### Patterns of Contraceptive Use Amongst Study Participants

**Fig. 1:**
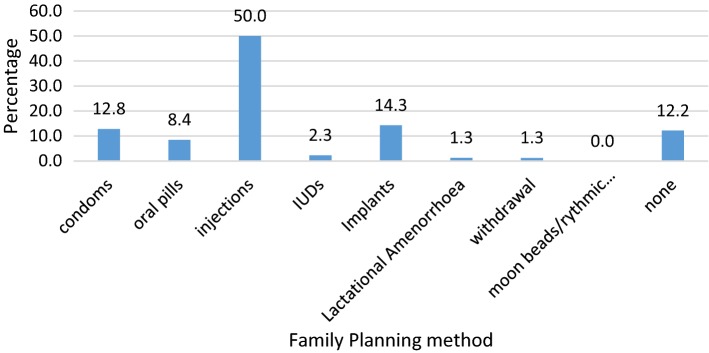
Previous history of contraceptive use

All women (100%) were aware of family planning services at MRRH. A total of 278 (87%) had ever used any form of modern family planning method, only 121 (38%) of women used it in the last 2 years and 22 (8%) women reported dual contraception (Table 1). Injectable family planning methods were the most-frequently used (50%) (Fig. 1). As shown in Table 2, 14% reported that their sexual partners disapproved of the use of contraception. Up to 78% of study participants who had used contraception reported that their sexual partners knew they were on family planning. Of the 278 (87%) women who had ever used modern family planning for at least a year, 226 (81%) stopped contraception because they had personal desire while 116 (42%) reported partner desire to have another child. Others reported stopping contraception due to inconvenience (16%), heavy bleeding (17%), or perceived conflict with religion (20%).

**Table 2:** Patterns of contraceptive use and reproductive health goals amongst study participants, N = 320

Characteristic	Frequency (%)
*Contraception*	
Ever used any modern family planning method	278 (86.9)
Used any modern family planning method in the last 2 years	121 (37.8)
Used condoms and any other modern family planning method together (n = 278)	22 (07.9)
My sexual partner disapproves of using contraception	46 (14.4)
My partner knew I was on family planning (n = 278)	217 (78.1)
*Pregnancy intentions*	
Partner wants to have another child in the next 2 years	186 (58.1)
I want to have another child in the next 2 years	175 (54.7)
Either partner wants to have another child in the next 2 years	189 (59.1)
I need to have as many children as my partner desires	110 (34.4)
Decision making on when to get pregnant is;	
Independent	102 (37.2)
I contribute	84 (30.7)
It is entirely up to spouse/sexual partner	88 (32.1)
How often participant ever discussed family planning with spouse/sexual partner	
0	24 (07.5)
1–2 times	105 (32.8)
3–4 times	67 (20.9)
> 4 times	124 (38.8)
I discontinued the last contraceptive use because, (n = 278);	
I wanted to have another child	226 (81.3)
My spouse wanted to have another child	116 (41.7)
It was inconvenient	45 (16.2)
Heavy bleeding	55 (19.8)
My religion forbids use of contraceptive use or against God’s plan	55 (19.8)
Others (my spouse was away, expensive)	17 (06.1)
Reasons for not wanting to become pregnant again (n = 140)	
Financial	42 (30.0)
Health	39 (27.9)
Have enough children	54 (38.6)
Other	5 (03.6)
Reasons for future pregnancy desire (n = 175)	
Few children	37 (21.1)
Sex preference	30 (17.1)
Death of a child	9 (05.1)
Partner desires more children	83 (47.4)
No response	16 (09.1)

### Pregnancy Intentions

One hundred and seventy five (55%) women reported desiring more children in the next two years. In addition, over half (N = 186, 58%) of women reported that their sexual partners/spouses wanted more children in the future. One-hundred and eighty nine (59%) of women reported either personal or partner desire for more children in the next 2 years. Eighty eight (32%) of women described that the decision on when to get pregnant entirely depended on their spouse/sexual partners with 110 (34%) women reporting that they will have as many children as their sexual partners’ desire. Reasons for future pregnancy desires ranged from sex/gender preference (N = 30, 17%), cultural expectations for more children (N = 37, 21%), partner desires for another child (N = 183, 47%) and death of a child (N = 9, 5%). Of the study participants who did not want to become pregnant again, 39% (N = 54) felt they had enough children, 30% (N = 42) and 28% (N = 39) were due to financial and health reasons respectively.

### Factors Associated with Pregnancy Intentions

In bivariate analyses, several factors were associated with pregnancy intention (Table 3). In the multivariate model, increasing age (AOR = 0.34, P = 0.012) and previous use of modern contraception for at least a year (AOR = 0.07, P = 0.001) were associated with decreased desire to have another child in the next two years. Increasing parity of more than two (AOR = 0.59, P = 0.015) and having more than two biological children less than 18 years of age and still living in her household (AOR = 0.42, P = 0.021) were associated with decreased adjusted odds of pregnancy intention. Parity and number of own children living in a household seem different as some pregnancies reported and or children were lost.

**Table 3:** Factors associated with report of pregnancy intentions in the next 2 years postpartum

Characteristic effect estimate	Univariable analysis		Multivariable analysis			
	Crude odds ratio	P	Adjusted odds ratio	P	Adjusted odds ratio^a^	P
Participant age	0.19 (0.11, 0.35)	0.000	0.34 (0.14, 0.79)	0.012	0.48 (0.24, 0.94)	0.033
Partner age	0.36 (0.21, 0.61)	0.000	N/A		N/A	
CD4 < 250 (yes/no)	0.66 (0.51, 0.85)	0.014	N/A^b^		N/A	
Duration of ART > 3 years	0.35 (0.20,0.61)	0.000	0.88 (0.72, 1.09)	0.054	0.47 (0.27, 1.10)	0.091
> primary education (yes/no)	1.39 (0.89, 2.18)	0.151	N/A		N/A	
Disclosed to sexual partner (yes/no)	1.45 (0.73, 2.88)	0.280	N/A		N/A	
Partner wants to have child in future	56.33 (31.75, 99.95)	0.000	31.36 (15.17,64.86)	0.000	N/A	
Planned referent pregnancy (yes/no)	3.97 (2.04, 7.76)	0.000	2.69 (0.99, 8.42)	0.050	2.34 (1.12, 4.93)	0.026
Alcohol consumption (yes/no)	1.25 (0.77, 2.01)	0.362	N/A		N/A	
Used modern FP method^c^ (yes/no)	0.40 (0.18, 0.72)	0.000	0.07 (0.01, 0.34)	0.001	0.25 (0.09, 0.70)	0.008
Used natural FP methods^d^ (yes/no)	2.42 (0.25-23.70)	0.432	N/A		N/A	
Parity ≥ 3	0.08 (0.03, 0.36)	0.000	0.59 (0.39, 0.90)	0.015	0.66 (0.45, 0.97)	0.035
≥ 3 alive, own children in household	0.27 (0.17, 0.45)	0.000	0.42 (0.19, 0.94)	0.021	0.61 (0.42, 0.87)	0.007
≥ 2 non-biological children in household	0.82 (0.42, 1.61)	0.571	N/A		N/A	
Last 2 deliveries < 2 years	0.51 (0.32, 0.81)	0.004	0.71 (0.41, 1.65)	0.232	0.97 (0.75, 1.25)	0.802
Prenatal visits > 2	1.18 (0.74, 1.89)	0.489	N/A		N/A	
At least 1 adult working in household	1.29 (0.76, 2.19)	0.350	N/A		N/A	
Household income > 150,000	1.82 (1.08, 3.05)	0.002	1.37 (1.08, 1.75)	0.010	1.21(1.04, 1.38)	0.001
Monogamous household	2.0 (0.59, 6.74)	0.254	N/A		N/A	
Receives social support	1.69 (1.03, 2.75)	0.034	1.64 (0.83, 3.22)	0.075	1.73 (0.89, 2.90)	0.081
Depression	0.36 (0.17, 0.75)	0.004	0.63 (0.23, 1.74)	0.068	0.42 (0.18, 1.06)	0.113
Stigma	1.09 (0.66, 1.81)	0.733	N/A		N/A	
Food insecurity						
No (HFIAS ≤ 8)	Ref					
Yes (HFIAS > 8)	0.42 (0.23, 0.76)	0.003	0.36 (0.17, 1.12)	0.072	0.45 (0.19, 1.07)	0.070

^a^Dropped partner desires to see effect of other variables on individual aspirations

^b^Collinear with duration on ART

^c^Includes contraceptive pills, male/female Condoms, diaphragm, cervical cap, Intrauterine Device (IUD), contraceptive implant, injectables, emergency contraception methods to limit or space the number of children

^d^Includes standard days and lactation amenorrhea method

Partner pregnancy intention was strongly associated with increased adjusted odds of pregnancy intention (AOR = 31.36, P < 0.000), as was reporting her most recent pregnancy as planned (AOR = 2.69, P = 0.050), and having a higher household income per month (AOR = 1.37, P = 0.010).

The findings are similar in a second model when the partner’s desire to have children is dropped/muted.

## Discussion

Among adult postpartum WLWH in southwestern Uganda, we found that 59% report personal or partner desire for another child in the next 2 years. In a rural setting where fertility rates are among the highest in the world, intent to have more children was strongly associated with perceived partner desire for more children, previous planned pregnancy, and higher household income. Previous use of modern contraception, increasing age, parity of three or more and having more than two children living in the household were independently associated with reduced odds of reporting intent to have another child in the next 2 years. This analysis highlights the importance of comprehensive family planning that addresses fertility desires and the key role played by male partners, household income, parity and previous exposure to modern contraception among WLWH.

The importance of male partners in influencing reproductive decision-making has been described. Generally, and regardless of serostatus, male partners have been shown to have great influence on having another child, but receive minimal counselling about the benefits of spacing, options for safer pre and conception practices [8, 25–27]. Nieves and colleagues also noted that partner’s fertility desire was independently associated with women’s contraceptive use [3]. In our study, perceived male partner desire for future pregnancy was associated with a 31-fold increased odds in female plans for pregnancy. It was also noted that over 99% of the women who reported their own desire to have more children also reported their partner’s desire to have more children. However, a study by Snow and colleagues, published in 2013 based on data from rural Uganda, reported that marriage partners of WLWH were less likely to want more children than partners of HIV-negative women, with WLWH more likely to report discordant preferences for no more children even when their partners wanted more kids [1]. These findings were noted regardless of whether the women were on ART. In our study however, all women were enrolled on ART, with mean ART duration and CD4 count of 4.5 years [SD = 3.9] and 395 cells/mm^3^ [SD = 62] respectively. This overwhelming success of eMTCT programming and treatment in Uganda and possible reduction in stigma for PLWH who want to have children may explain the higher percentage of WLWH reporting desires to have more children (54%) in our study compared to 28% reported by Snow and colleagues. The perceived partner desires for more children were also higher (58%). Such higher fertility intentions could further be attributed to lower stigma levels (28%), improved social support (median = 2.4, IQR = 2.1–2.8) and high HIV disclosure rate (88%) observed within this population, highlighting the importance of integrating comprehensive family planning care into HIV and antenatal, postnatal and paediatric care for WLWH and their families.

Men in Uganda have often been regarded as unsupportive of their partner’s use of family planning [28] and some studies show that poor communication within partnerships may make it challenging for men to be involved [8]. In our study, 78% of the women reported that their partners knew they were on family planning, the rest choosing not to disclose it, while 14% of the sexual partners reportedly disapproved their partners using contraception. Half of the women who reported previous use of family planning used injectables. Some studies have shown that providing men with supportive opportunities to access information on safer conception practices by health care providers is key in influencing their pregnancy intentions, attitude and uptake of reproductive health services [27, 29]. Their active involvement in reproductive health has been facilitated by a clear understanding and integration of the existing gender-based and socio-cultural norms into routine sexual and reproductive health programs [30]. This approach according to Onyango and colleagues makes services more male-friendly, while helping to debunk negative traditional beliefs surrounding reproductive health practices thus supporting the health of men and women and families. Training of male peer trainers and mobilisers has also been found to improve contraceptive uptake [31]. Other attempts to actively involve men in reproductive health and maternity care have shown to improve their decision making, presence during antenatal visits, postpartum visits, childbirth, and the initiation of breastfeeding within 1 h of birth, leading to better maternal and new born outcomes [32, 33]. These carefully thought out strategies to actively engage male partners in sexual and reproductive programs, coupled with improved access to appropriate information and effective contraception could therefore be crucial to realising positive outcomes championing better contraceptive uptake to prevent unintended/unplanned pregnancies amongst WLWH in Uganda. The couple’s improved awareness of their health risks, contraception use benefits and the available HIV prevention and conception strategies could also guide and influence their decision on when or if to have another child in the future. Indeed in our adjusted model, previous use of modern contraception was independently associated with reduced pregnancy intentions within 2 years postpartum, alongside increasing age, parity or children living in a particular household. Additionally, the higher rate of planned pregnancies (83%), coupled with a high pregnancy desire observed in this population emphasise the vital need to counsel WLWH and their spouses not only about contraception but also opportunities for safer conception. The observed high rate of injectables on the other hand shows how much women rely on this method and the importance of understanding the reasons for its preference as well as the effects of ART on levels of hormonal therapy.

The impact of HIV on pregnancy intentions is affected by underlying fertility norms and beliefs about the importance of child bearing in a society with or without ART [34, 35]. In Uganda, the key role of boy child bearing and number of children born for example, has been well documented [35]. In this study, factors such as bearing of own children or having a considerable number of children (at least 3) < 18 years of age living in a particular household or increasing parity were indeed associated with suppressed pregnancy intentions alongside age within this study population. These findings are in line with previous studies showing decreased fertility aspirations with the economic burden of caring for children below 18 years of age in a household [1, 36]. However, a person’s ability to generate regular or more income, overcome barriers to provide basic needs like food, shelter, medicines and instrumental social support has been found to positively-impact households of persons living with HIV [37, 38]. This eased economic burden and improved quality of life may therefore explain a key role played by increasing household incomes in predicting women’s greater interest in having more children in future observed in this study.

Food security and social support received, which seemed to significantly influence pregnancy desires were edged out in a regression model probably due to their known interdependent association with adherence among people living with HIV [38]. The low CD4 count < 250 cells/mm^3^ and duration on ART also seemed important in diminishing pregnancy intentions within 2 years postpartum in unadjusted model but looked to be greatly dependent on other factors like food security, social support, partner desires and contraception use, which edged them out in the multivariate analysis.

## Strengths and Limitations

This work presents baseline data for an ongoing randomized controlled trial at a publicly-funded and operated hospital in a rural low-resource setting that delivers over 12,000 women annually from various social and demographic backgrounds, making results generalizable to similar settings. The population of postpartum WLWH with a high proportion of pregnancy intentions also enabled us to document the effect of age, duration on ART, CD4 count, disclosure, parity, living with children in a homestead, perceived spousal pregnancy desires, planned pregnancy, household income, prior use of modern contraception and mode of referent delivery within a multivariate model.

There were also limitations to this work. Individual and perceived spousal desires to have more children were both reported by the study participant. There is therefore a possibility that assumptions about partners desiring more children was skewed depending on the structural or cultural underpinnings/understanding of the roles and women’s identity of child bearing with in this setting. There may also have been an under report of pregnancy intentions due to stigma of WLWH and fear of being reprimanded by health care providers especially immediately after child birth. Women may also be less interested in subsequent child bearing within a few days postpartum.

## Conclusion

Our findings highlight the striking role male partners play in influencing pregnancy intentions postpartum alongside income, planned pregnancy, age, parity and previous contraceptive use amongst WLWH in modern Uganda. In adjusted analysis, perceived male partner desire for future pregnancy was associated with a 31-fold increased odds in female plans for pregnancy. It is therefore very important to accept gender norms in this context, actively engage male partners in sexual and reproductive health counselling about child spacing for the health of women, children, and families alongside individual-level social, economic and structural factors within which couples can ably and supportively understand the risks of unplanned pregnancies and thus access reliable contraceptive methods whenever they need or want them. The high rate of planned referent pregnancy and future plans for pregnancy noted in this population of WLWH on ART requires integration of these reproductive health services into routine HIV care, which could also improve access to contraception and minimise the burden (time and money) spent looking for such services by WLWH. Additionally, the observed high rate of injectables shows how much women rely on this method and the importance of understanding the effects of ART on levels of hormonal therapy.
